# Changes in oral microbiota due to orthodontic appliances: a systematic review

**DOI:** 10.1080/20002297.2018.1476645

**Published:** 2018-07-03

**Authors:** Alessandra Lucchese, Lars Bondemark, Marta Marcolina, Maurizio Manuelli

**Affiliations:** a Department of Orthodontics, Vita Salute San Raffaele University, Milan, Italy; b Unit of Dentistry, Division of Orthodontics, Research area in Dentofacial Orthopedics and Orthodontics, IRCCS San Raffaele Scientific Institute, Milan, Italy; c Department of Orthodontics, Faculty of Odontology, Malmő University, Malmő, Sweden

**Keywords:** Oral microbiota, biomaterial science, orthodontic appliances, periodontopathic bacteria, caries bacteria

## Abstract

**Background**: Oral microbiota has been at the center of cultural attention in recent years. In daily clinical practice, orthodontic appliances may be associated with an increased cariogenic risk and a worsening of preexisting periodontal diseases.

**Objective**: The purpose of this review is to investigate the available evidence regarding the association between orthodontic appliances and changes in the quality and quantity of the oral microbiota.

**Design**: The research included every article published up to October 2017 featuring the keywords ‘Orthodontic appliance* AND (microbiological colonization OR periodontal pathogen* OR *Streptococcus mutans* OR *Lactobacillus* spp. OR *Candida* OR *Tannerella forsythia* OR *Treponema denticola* OR *Fusobacterium nucleatum* OR *Aggregatibacter actinomycetemcomitans* OR *Prevotella intermedia* OR *Prevotella nigrescens* OR *Porphyromonas gingivalis*)’ and was conducted in the major medical databases. The methodological quality of selected papers was scored using the ‘Swedish Council on Technology Assessment in Health Care Criteria for Grading Assessed Studies’ (SBU) method.

**Results**: Orthodontic appliances influence the oral microbiota with an increase in the counts of *S. mutans* and *Lactobacillus* spp. and in the percentage of potentially pathogenic gram-negative bacteria.

**Conclusions**: There is moderate/high evidence regarding the association between orthodontic appliances and changes in the oral microbiota.

PROSPERO registration number CRD42018091589.

## Introduction

Periodontal health is crucial and requires special attention when performing an orthodontic treatment plan, both in adult and pediatric patients [1]. Preserving the integrity of periodontal tissues is one of the main concerns of orthodontics specialists, which has led to the definition of specific hygiene protocols for orthodontic patients [2]. Since 1985, the scientific community has been very concerned about the interaction between orthodontic devices and oral bacteria [3,4]; in fact, the first studies to analyze the oral microbiota and conventional braces (CB) took place in this period. In 2012, Freitas et al. published a systematic review regarding the alteration of the oral microbiota caused by fixed appliances [5]. The authors concluded that ‘The literature revealed moderate evidence that the presence of fixed appliances influences the quantity and quality of oral microbiota’. However, the authors included papers that analyzed bacteria from appliance surfaces and from oral mucosa, without distinction.

Furthermore, a significant number of studies have been published since 2012. Our review aims to update the research of Freitas et al., focusing on studies that have analyzed the microbiota collected from oral sites and not directly from appliances, and including all appliance types (self-ligating braces, invisalign aligners, sports-mouthguards, and other removable appliances) and not only fixed appliances.

Thus, the clinical research questions were as follows:

- Do orthodontic appliances influence the quality and quantity of the oral microbiota?
- What are the effects of orthodontic devices on the different bacterial species in the oral cavity?

## Materials and methods

A search of the keywords Orthodontic appliance* AND (microbiological colonization OR periodontal pathogen* OR *Streptococcus mutans* OR *Lactobacillus* spp. OR *Candida* OR *Tannerella forsythia* OR *Treponema denticola* OR *Fusobacterium nucleatum* OR *Aggregatibacter actinomycetemcomitans* OR *Prevotella intermedia* OR *Prevotella nigrescens* OR *Porphyromonas gingivalis*) was conducted in PubMed, PMC, Scopus, Lilacs, Scielo, Cochrane Trial Library, Web of Science. All articles published up to October 2017 were included. The Preferred Reporting Items for Reporting Systematic reviews and the Meta Analyses protocol were adopted for this systematic review [6].

**Figure 1.**
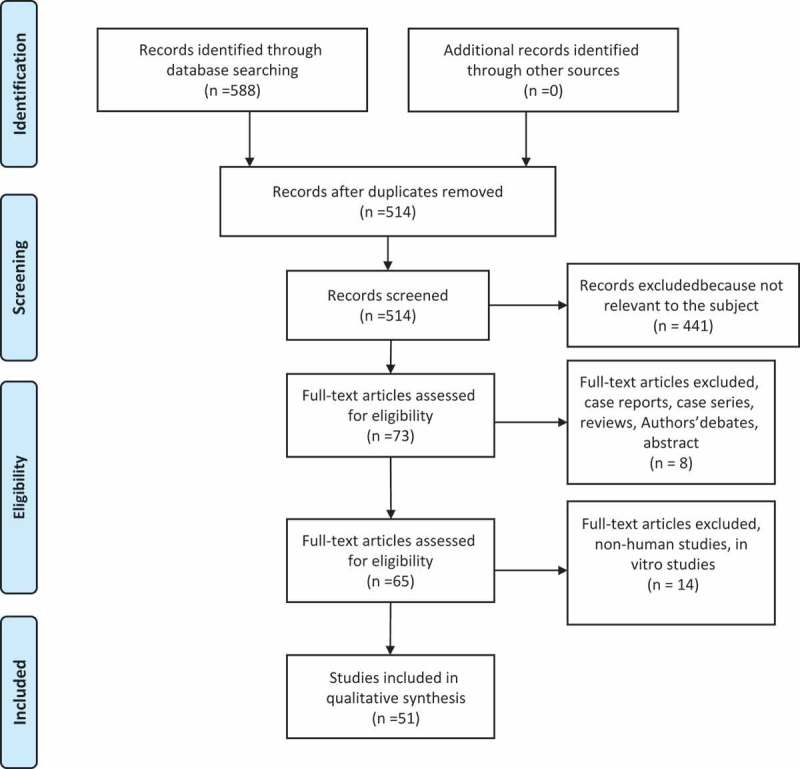
Article selection process.

During the first phase, all the articles were selected by title and abstract by two of the authors and duplicate exclusion was performed. In the next phase, the full texts of potentially relevant papers were evaluated to determine if they met the eligibility criteria. Articles were selected on the basis of the criteria listed in Table 1. The article selection process is illustrated in Figure 1. Discussions were held to resolve any disagreements; when a resolution could not be found, a third review was consulted. Data extraction from the selected papers was performed independently by two review authors who adopted a template similar to that of Freitas et al. [5]. The template was adapted to the necessities of our study and is shown in Table 2 [5].

**Table 1.** Study selection criteria.

Inclusion criteria	Exclusion criteria
Trials analyzing quantity and/or quality of oral microbiota on oral surfaces of orthodontic patients At least 10 patients analyzed At least two time points for analysis (with at least one before the beginning of treatment) Statistical analysis of results	Absence of baseline investigation before appliances placament Comparing microbiota only among different patients and not longitudinally in the same group Patients with systemic diseases Antibiotic therapy 3 months before and during the study No standardization and training in oral hygiene Use of mouth rinse during investigation *In vitro* or animal studies Case reports, case series, reviews, author opinions

**Table 2.** Characteristics of studies included in the review.

		Participants			Collection time								
Reference	Study design	Sample size (male/female)	Groups	Age	Appliance	T0	T1	T2	T3	T4	Collection methods	Microbial analysis methods	Quality of the study
Al-Anezi [9]	RCT (cross-arch)	24	1	Mean: 12.6 years ± 1.01 month	SL braces + elastomeric modules	Before bonding	3 months				Sterile paper points from the lateral incisors ligated with and without elastomeric modules	PCR + DGGE	B
Alves et al. [10]	RCT (split mouth)	14 (6 M/8 F)	1	Mean: 17 years ± 2.6 months	CB/Steel ligatures vs. CB/elastomeric rings	Before bonding	6 months				Sterilized periodontal curette 2 mm supragingival and 2 mm subgingival	PCR	B
Arab et al. [12]	Prospective study	30 (6 M/24 F)	1	12–18 years	CB	Before bonding	6 weeks	12 weeks	18 weeks		Saliva collected by spitting into a sterile test tube for 10 min	Number CFU/ml was quantified	B
Arikan et al. [11]	RCT	38 (20 M/18 F)	2 G1 (fixed): 9 M/10 F G2 (removable): 11 M/8 F	4–10 years	Fixed and removable space maintainers	Before appliance of maintainers	1 month	3 months	6 months		Sterile foam pads soaked in Sabouraud’s broth on six mucosal surfaces	*Candida*: colonies were counted separately for each site by visual examination and expressed as CFU/mm^2^ *E. faecalis*: counted macroscopically based on characteristic gram-stain morphology and recorded as CFU/ml of the original saliva sample	B
Arslan et al. [13]	Prospective study	42 (23 F/19 M)	1	Mean: 19.8 years	CB	1 month before bonding	1 month	6 months	12 months		Samples taken from saliva, enamel surfaces of U5 and L5, and U1, and L1 adjacent to the braces with sterile wooden toothpicks (at T0 samples only from saliva and not from the teeth)	*Candida* identiﬁed by gram-staining, a germ-tube test, chlamydospore, and an API 20C AUX system (Bio-Mérieux, Marcyl’Etoile, France). *Candida* colonies on the plates were counted	B
Baka et al. [14]	RCT (split mouth)	20 (20 M)	2 G1: SL braces G2: CB	Mean: 14.2 years ± 1.5 months	SL braces vs. CB/steel ligature	Before bonding	1 week	3 months			Sterilized curettes from the labial surfaces of U2 and L2 left and right	DNA extracted from supragingival plaque samples (Dneasy blood and tissue kit) + real-time PCR	B
Demling et al. [15]	Prospective study	10 (8 F/2 M)	1	Mean: 29.0 years ± 4.7 months	Lingual braces	Before bonding	3 months				Samples of gingival crevicular ﬂuid taken using sterile paper points. Buccal and lingual sites of U6 and L6, U4 and L4, U1 and L1. In extraction cases, the U5 and L5 instead of the U4 and L4	PCR	B
Demling et al. [16]	Prospective study	20 (6 M/14 F)	1	Mean: 22.3 years ± 8.6 months	Lingual braces	Before bonding	4 weeks				Gingival crevicular fluid taken with sterile paper points at labial and lingual sites of U6 and L6, U4 and L4, and U1 and L1. In extraction cases, U5 and L5 instead of U4 and L4	DNA extracted with a QIAmp DNA Mini Kit + PCR	B
D’Ercole et al. [17]	Prospective study	60 (27 M/33 F)	1	Mean: 9.9 years ± 1.2 months	Sport mouthguards	Before mouthguard	6 months	1 year	6 months without		Stimulate saliva with paraffin wax to chew and saliva collected for 5 min in a measuring cup	CFUs of SM counts per milliliter of saliva (CFU/ml) GC saliva-check mutans (GC Corp., Belgium)	B
Farhadian et al. [18]	RCT	66	2 G1: Conventional removable retainers G2: Removable retainers containing silver nanoparticles	Age ≤25 years	Conventional removable retainers vs. removable retainers containing silver nanoparticles		1 week after debonding	7 weeks after retainer delivery			Swab samples were taken from the maxillary palatal side	Number of SM CFU was counted with a digital colony counter	B
Forsberg et al. [19]	RCT (splith mouth)	12 (6 M/6 F)	1	12–14 years	Ligature wires vs. elastomeric rings (CB)	1 week before bonding	Before bonding	4 weeks	10 weeks	T4: 19 weeks T5: 34 weeks T6: 61 weeks T7: 6 weeks after removal	Stimulated saliva samples, plaque samples collected with charcoaled points from U2	CFU count of SM and LB	B
Ghijselings et al. [21]	Prospective study	24 (10 M/14 F)	2 G1: 10 (4 M/6 F) braces only G2: 14 additionally treated with a headgear	Mean: 14.6 years ± 1.1 months	CB vs. CB + headgear	Tb G1: Bonding time G2: 18 weeks before G1	Braces removal	3 months follow-up	2 years follow-up		Supragingival plaque: removed by means of sterile curettes. Subgingival plaque: six sterile medium paper points inserted per site (three mesially and three distally) and kept in place for 10 s	Total number of, respectively, anaerobic and aerobic CFU was counted. Speciﬁc black-pigmented colonies on a nonselective anaerobic plate were counted	B
Hägg et al. [22]	Prospective study	27 (13 M/14 F)	1	Mean: 15.5 years ± 2.3 months	CB	Before bonding	Examined 3 times during a 3-month follow-up	Examined 3 times during a 3-month follow-up	Examined 3 times during a 3-month follow-up		Imprint culture: sterile plastic foam pads dipped in Sabouraud’s broth and placed on the dorsum of the tongue. Oral rinse. Pooled plaque	Imprint culture Candidal: visual counting CFU Yeats: gram-stain, germ tube test, and the API 20C AUX assimilation test Oral rinse: *Candida* and *Enterobacteriaceae*: CFU Pooled plaque: *Candida* and *Enterobacteriaceae*: CFU	C
Hernández-Solis et al. [23]	Prospective study	60	1	4–10 years	Orthodontic appliance	Before appliance	6 months				Samples taken with a sterile swab rubbed over: oral mucosa and the back of the tongue	PCR	C
Ireland et al. [24]	RCT (split mouth)	24	1	11–14 years	SL braces + bands + bonded molar tubes to contralateral quadrants of the mouth + elastomeric ligature on one U2 bracket	Pre-bond-up at the molar separator appointment	3 months	Just prior of debonding	3 months post-debond	1 y post-debond	Supragingival plaque samples: on molars (bands and bonds) using sterile curettes and subgingivally using sterile paper points U2 (with or without elastomeric ligation): supragingival plaque collected adjacent to the bracket margins	PCR + microarray hybridization	B
Jurela et al. [25]	Prospective study	32	2 G1: 16 CB G2: 16 esthetic braces	13–30 years	CB vs. esthetic plastic braces	Before bonding	12 weeks				Saliva samples	PCR + cultivation method	B
Kim et al. [26]	Prospective study	30	1	Mean: 16.7 years ± 6.5 months	CB	Before bonding	1 week	3 months	6 months		Sterile paper points from the distobuccal gingival crevice of the left U1, the left L1, the mesiobuccal gingival crevice of the left U6, and the left L6	PCR	B
Kupietzky et al. [27]	Prospective study (case control)	64	2 G1: 32 braces G2: 32 control	12–15 years	CB	G1: Before bonding G2: 2 months before G1	2 months				Salivary collection and bacterial culture followed manufacturer’s instructions	LB and SM CFU were compared with standard densities	C
Lara-Carrillo et al. [28]	Prospective study	30 (11 M/19 F)	1	M mean: 16.5 years ± 3.7 months F mean: 16.5 years ± 5.5 months	CB	Before bonding	1 months	Canine retraction (placement of elastic chain in mouth)	Anterior segment retraction (placement of closing loops in mouth)		Buccal surface of U6, collected with a Q-tip	Dentocult-SM + Dentocult-LB	B
Lara-Carrillo et al. [29]	Prospective study	34 (14 M/20 F)	1	Mean: 16.7 years ± 5.2 months	CB	Before bonding	1 month				Unstimulated saliva from inner mucosa Stimulated saliva by chewing Sterilized cotton swab on U6	SM: Dentocult® SM LB: Dentocult® LB	B
Leung et al. [30]	Prospective study	27 (14 M/13 F)	1	Mean: 14.9 years	CB	Before bonding	At least 4 weeks after (mean 7 weeks)				BEC: sterile cytologic brushes on both cheeks. Plaque samples were obtained on the buccal surfaces of the 4-s premolars. Supragingival and subgingival plaque: removed with a sterile periodontal curette	PCR + FISH	C
Levrini et al. [31]	RCT	77 (52 F/25 M)	3 G1: Invisalign G2: CB G3: Control	Mean: 24.3 years	Invisalign CB	Begin of the teatment	1 month	3 months			Sterile paper point into the deepest part of the gingival sulcus for 30 s. Sites: U6 right (Site 0) and U1 left (Site 1)	Real-time PCR	B
Liu et al. [32]	Prospective study	17	1	Mean: 12.6 years	CB	Before bonding	1 month	3 months	6 months		Sterile probe passed along the supragingival smooth surface of the upper right teeth	Levels of total viable count, total Streptococci and SM in dental plaque + AP-PCR	B
Lombardo et al. [33]	RCT	20 (15 F/5 M)	2 G1: 10 (8 F/2 M) CB G2: 10 (7 F/3 M) lingual braces	G1: Mean: 19.3 years ± 3.6 months G2: Mean: 22.3 years ± 3.2 months	CB vs. lingual braces	Before bonding	4 weeks	8 weeks			Stimulated saliva collected by chewing paraffin gum for 5 min and expectorating into a sterile cup	Colonies were counted	B
Maret et al. [34]	Prospective study (case control)	95 (56 F/39 M)	2 G1 (32 F/16 M): 48 CB G2 (24 F/23 M): 47 control	12–16 years	CB vs. control	Before bonding	6 months				Stimulated saliva samples: chewing paraffin wax until 2 ml of saliva had been collected	Salivary SM and LB: Dentocult® SM strips and Dentocult® LB method	C
Mattingly et al. [3]	Prospective study	10 (6 M/4 F)	1	12–25 years	CB	T0–T1–T2: Three visits before bonding (distance of 10 days)	T3–T4–T5: Three visits after bonding (distance of 10 days)				Plaque samples collected with a sterile dental explorer between bracket base and the gingival margin	SM CFU count	C
Miura et al. [35]	RCT	40	2 G1: 20 Fluoride-releasing elastomeric ligature ties G2: 20 Conventional elastomeric ligature ties	12–20 years	Fluoride-releasing elastomeric ligature ties vs. conventional elastomeric ligature ties	Before ligature	Ligature	7 days	14 days	28 days	Saliva and plaque samples. A sterilized curette was used to collect plaque samples from the area surrounding the ligature ties of the right U2, left U5, left L3, and right L5	Number of SM CFU	B
Nalçacı et al. [36]	Prospective study	46 (14 F/22 M)	2 G1: 23 (11 F/12 M) SL braces G2: 23 (13 F/10 M) CB	11–16 years	SL braces vs. CB	Before bonding	1 week	5 weeks			Microbial samples taken from the buccal surfaces of all bonded teeth	Number of colonies determined under a stereomicroscope	B
Ortu et al. [56]	Prospective study	30 (15 M/15 F)	3 G1: 10 RPE G2: 10 McNamara expander G3: 10 Controls	6–9 years	RPE vs. McNamara expander vs. controls	Before initiation of expansion therapy	3 months	6 months			Whole stimulated saliva, stimulated with paraffin-based sticks	CFU of SM and LB	B
Pandis et al. [38]	RCT	32	2 G1: CB G2: SL braces	Mean: 13.6 years	CB ligated with conventional elastomeric modules vs. SL braces	Before bonding	2–3 months				Collect saliva in the mouth and to expectorate into a chilled empty petri dish approximately 3 ml of saliva	Salivary SM and total bacteria were enumerated and analyzed after growth in culture	B
Paolantonio et al. [39]	Prospective study (splith mouth)	24 (11 M/13 F)	1	18–22 years	CB in one dental arch vs. control sites	Before bonding	4 weeks	8 weeks	12 weeks (removal)	4 weeks after removal	Supragingival plaque: sterile curette subgingival plaque: insertion of three sterile paper points at the deepest part of each gingival sulcus. Sites: mesiobuccal sites of U6–L6 and distobuccal sites of U2–L2 in both dental arches	Agar plates examined for presence of Aa. Definitive identification made on the basis of the methods: Gram-stain, nitrate reduction, production of catalase, urease and indole, growth on MacConkey agar, and fermentation reactions to carbohydrates	B
Pejda et al. [40]	RCT	38 (13 M/25 F)	2 G1: 19 (7 M/12 F) SL braces G2: 19 (6 M/13 F) CB	Mean: 14.6 years ± 2.0 months	SL braces vs. CB	Before bonding	6 weeks	12 weeks	18 weeks		Subgingival plaque samples were obtained at 18 weeks (T3). Supragingival plaque: removed with a probe. Subgingival plaque: collected with a sterile paper point from the periodontal sulcus. Sites: U6 right, U1, U4 left, L6 left, L1 right, L4 left	Micro-Dent test+ PCR	B
Pellegrini et al. [41]	RCT (split mouth)	14 (12: full appliances, 2: on maxillary arch only)	1	11.7–17.2 years	SL braces vs. CB with elastomeric ligatures	Before bonding	1 week	5 weeks			Plaque specimens collected from labial surfaces surrounding the brackets of U2 and L2 with a sterilized dental scaler. Saliva collection: chewing gum-shaped paraffin wax tablet chewed for 1–5 min	Total oral Streptococci: mitis salivarius agar Bacterial count Determination of ATP-driven bioluminescence with the Bac-Titer Glo Microbial Cell Viability Assay Kit	A
Perinetti et al. [42]	Prospective study	21 (11 F/10 M)	1	Mean: 17.1 years ± 3.3 months	CB	Before bonding	28 days				Subgingival plaque and GCF: three 30 standardized sterile paper strips inserted 1 mm into the gingival crevice. Mesial and distal tooth sites: U3 test (DC), its contralateral (CC), and antagonist (AC) used as controls. CC included in the orthodontic appliance, but not subjected to the orthodontic force; AC free from any appliance.	Aa colonization was determined by culture methods, while ALP and AST activities were evaluated spectrophotometrically	B
Peros et al. [43]	Prospective study	23	1	12–17 years	CB + bands + wire ligatures	Before bonding	6 weeks	12 weeks	18 weeks		Chewed bilaterally a piece of paraffin wax	Salivary analysis: Cultura incubator and a CRT bacteria test kit for SM and LB	B
Ristic et al. [44]	Prospective study	32 (13 M/19 F)	1	12–18 years	CB + bands	TX: First appointment T0: 3 weeks before bonding	1 month	3 months	6 months		Two sterile paper points in to the deepest part of gingival sulcus. Sites: mesio-vestibular points of subgingival sulcus of: U6 right, U1 left, and U4 left. If one was missing, adjacent tooth from the same group was used	Semiquantative determination of anaerobe colonies using direct counting and density comparison. Subculturing, gram-stain, and identification tests of biochemical reactions: for identification of bacteria species	B
Ristic et al. [45]	Prospective study	32 (13 M/19 F)	1	12–18 years	CB + bands	Before bonding	1 month	3 months	6 months		Two sterile paper points in to the deepest part of gingival sulcus. Sites: mesio-vestibular points of subgingival sulcus of: U6 right, U1 left, and U4 left. If one was missing, adjacent tooth from the same group was used	Colonies of bacteria were counted. Subculturing, gram-stain, and identiﬁcation tests of biochemical reactions: used for identiﬁcation of bacterial species	B
Sfondrini et al. [46]	RCT (split mouth)	20 (6 M/14 F)	1	Mean: 23.8 years	Buccal and lingual braces (same braces) vs. control	Before bonding	1 day	7 days	30 days		Microbiological samples from the brackets and the teeth. Supragingival dental plaque: sterile curettes	Total CFU, SM CFU, and anaerobe CFU were measured	B
Shukla et al. [47]	RCT	60	1	15–25 years	CB	Before bonding	2 months	3 months			Plaque samples collected with sterile cotton swab sites: buccal and labial aspects of the anterior teeth and four first molars	Counts of SM were determined by using Dentocult SM kit	B
Shukla et al. [58]	RCT	60	1	13–18 years	CB	Before bonding	2 months	3 months			Plaque samples collected with sterile cotton swab sites: buccal and labial aspects of the anterior teeth and four first molars	Counts of SM were determined by using Dentocult SM kit *Candida* cultured on Sabouraud’s dextrose agar	B
Sinclair et al. [4]	RCT	13 (5 M/8 F)	1	Mean: 14 years ± 1 month	CB + bands	Before bonding	1 year				Subgingival plaque samples collected with a stainless steel orthodontic wire sites: U1–L1 and U6–L6 right	Mean counts for the triplicate plates of the five types of medium used	C
Sudarević et al. [48]	Prospective study	22 (12 F/10 M)	1	Mean: 25.09 years ± 4.36 months	CB + elastomeric ligatures	Before bonding	12 weeks				Chewing paraffin wax followed by saliva collection	PCR for SM and *S. sobrinus*	C
Thornberg et al. [49]	Prospective study	190 (47% M/53% F)	1	13.6 years	CB	Before bonding	6 months	12 months	>12 months	≈3 months after removal	Subgingival plaque samples: sterile paper points. Sites: Mesial U6 right, distal U1 right, mesial L6 left, distal l1 left, mesial L4 right (if extracted mesial L5)	DNA probe tecnique	C
Topaloglu et al. [50]	Prospective study	69 (31 F/38 M)	2 G1: 34 Removable G2: 35 CB	6–17 years	CB vs. removable appliance	Before appliance	1 month	3 months	6 months		Samples of unstimulated saliva	Numbers of CFU per plate were counted.	C
Torlakovic et al. [51]	Prospective study	20 (8 M/12 F)	1	Mean: 12 years ± 1 month	CB	Before bonding	4 weeks	3 months	5 months		Supragingival plaque samples collected using a sterile Gracey curette Sites: Labial surface of U1 right and left	PCR + HOMIM	C
Turkkahraman et al. [52]	RCT (split mouth)	21 (12 F/9 M)	1 (Two subgroups: G1: Elastomeric G2: Ligature wires)	Mean: 15.37 years ± 3.76 months	CB + elastomeric rings vs. ligature wires	Before bonding	1 week	5 weeks			Microbial samples from labial surfaces of U5	Colonies were counted under a stereomicroscope	B
Türköz et al. [53]	Prospective study	24	1	14–20 years	CB and thermoplastic retainers in the retention period	After debonding	15 days	30 days	60 days		Spit about 5 ml of saliva into 50-ml sterile tubes. Plaque samples collected with sterile swabs from gingival margin and enamel surface of each tooth at vestibule and palatal-lingual sides	Total viable SM and LB colonies were counted – means of CFUs per milliliter of volume (CFU/ml)	B
Uzuner et al. [54]	RCT	40 (29 F/11 M)	2 G1: 20 CB G2: 20 SL braces	14–16 years	CB + steel wire ligature vs. SL braces	Before bonding	1 month				Microbial samples were collected from the stimulated saliva and the plaque from the labial surfaces of the U2–L2, immediately surrounding the orthodontic brackets with a dental scaler	To estimate the number of CFUs of SM and LB, Dentocult SM and LB kits were used	B
Van Gastel et al. [20]	Prospective study	24 (10 M/14 F)	2 G1 (headgear): 14 (6 M/8 F) G2 (braces only): 10 (4 M/6 F)	Mean: 14.6 years ± 1.1 month	Headgear + bands + CB vs. CB	Tb G1: 18 weeks before G2 G2: Bonding time	T52 1 years				Periopaper absorbent strips into the sulcus for 30 s. The mesiobuccal and distobuccal sites of the U4 and U6 right were sampled. In the headgear group, U6 was banded, and U4 was bonded; the samples were analyzed separately	Total numbers of anaerobic and aerobic colony CFUs were counted. Pure cultures were identiﬁed by biochemical tests (including *N*-acetylb-d-glucosaminidase, α-glucosidase, α-galactosidase, α-fucosidase, esculine, indole, and trypsin activity)	B
Wichelhaus et al. [55]	Prospective study	11	1	Mean: 12.7 years	CB	Before bonding	4 weeks	12 weeks			Plaque removed from dental surfaces using a sterile curette. Sites: incisors, premolars and molars	PCR – 13C urea breath tests for HP – Dentocult® SM – Dentocult® LB	C
Zheng et al. [56]	RCT	50 (23 M/27 F)	1	Mean = 13.6 years	CB	Before bonding	1 month	2 months	3 months	6 months	Gargle method	Samples cultured in CHROMagar *Candida* identiﬁcation. Different *Candida* strains identiﬁed based on the color of the colonies + PCR	C

RCT: Randomized clinical trial; NS: non significant; SLB: self-ligating braces; PCR: polymerase chain reaction; DGEE: denaturing gradient gel electrophoresis; FISH: fluorescent *in situ* hybridization; AP-PCR: PCR with arbitrary primers; CFU/ml: colony-forming units per milliliter; Aa: *Aggregatibacter actinomycetemcomitans*; Tf: *Tannerella forsythia*; Pg: *Porphyromonas gingivalis*; Pi: *Prevotella intermedia*; Pn: *Prevotella nigrescens*; SM: *Streptococcus mutans*; LB: *Lactobacillus* spp.; HP: *Helicobacter pylori*; FOA: fixed oral appliance; U1: upper central incisor; L1: lower central incisor; U2: upper lateral incisor; L2: lower lateral incisor; U3: upper canine; L3: lower canine; U4: upper first premolar; L4: lower first premolar; U5: upper second premolar; L5: lower second premolar; U6: upper first molar; L6: lower first molar; FMPS: full-mouth plaque score; FMBS: full-mouth bleeding score; V: vestibular; L: lingual; HOMIM: Human Oral Microbe Identiﬁcation Microarray; RPE: rapid palatal expander.

Extracted data included first author, year of publication, study design, sample size, age of the patients, type of appliance analyzed, collection time of the study, collection methods, microbial analysis methods, and quality of the study.

### Quality analysis

The methodological quality is ‘the extent to which the design and conduct of a study are likely to have prevented systematic errors (bias)’. Variation in quality can explain variation in the results of studies included in a systematic review. More rigorously designed (better ‘quality’) trials are more likely to yield results that are closer to the ‘truth’ [7].

The methodological quality of selected papers was scored using the ‘Swedish Council on Technology Assessment in Health Care Criteria for Grading Assessed Studies’ (SBU) method, which was also used to assess the level of evidence for the conclusions of this review. The SBU method divided the methodological quality of the articles into three grades: grade A – high value of evidence, grade B – moderate value of evidence, and grade C – low value of evidence; once a score had been assigned to each study, the review’s level of evidence was stated in four grades: grade 1 – strong scientiﬁc evidence (at least two studies assessed at level A), grade 2 – moderate scientiﬁc evidence (one level A study and at least two studies at level B), grade 3 – limited scientiﬁc evidence (at least two studies at level B), and grade 4 – insufﬁcient scientiﬁc evidence (fewer than two studies at level B) (Table 3–4) [8].

**Table 3.** Swedish council on technology assessment in health-care (SBU) criteria for grading assessed studies.

SBU criteria for grading assessed studies
Grade A High value of evidence. All criteria should be met: randomized clinical study or a prospective study with a well-defined control group, defined diagnosis and end points, diagnostic reliability tests and reproducibility tests described, blinded outcome assessment
Grade B Moderate value of evidence. All criteria should be met: cohort study or retrospective case series with defined control or reference group, defined diagnosis and end points, diagnostic reliability tests, and reproducibility tests described
Grade C Low value of evidence. One or more of the conditions below: large attrition, unclear diagnosis, and end points, poorly defined patient material

**Table 4.** Definitions of evidence level.

Level	Evidence	Definition
1	Strong	At least two studies assessed with level ‘A’
2	Moderate	One study with level ‘A’ and at least two studies with level ‘B’
3	Limited	At least two studies with level ‘B’
4	Inconclusive	Fewer than two studies with level ‘B’

## Results

From the initial 588 articles, 51 were selected [3,4,9–57].

### Quality of evidence

In 37 of the 52 articles presented with moderate methodological quality [9–21,24–26,28,29,31–33,35–39,41–46,51–53,56,57], the major concern was the absence of repeatability tests. One article had a high quality [40] and the remaining 13 papers were classified as having a low quality [3,4,22,23,27,30,34,47–50,54,55]. Due to the lack of homogeneity in the study settings, a meta-analysis could not be applied and a systematic review realized.

### CB

Of the 29 articles that studied CB [3,4,10,12,13,19–23,26–30,32,34,35,38,41,44,46–48,50,51,54,44,57], the majority showed a significant increase in BOP and PI. Two studies [10,52] investigated the differences between the use of elastomeric or steel ligatures, revealing contradictory results on BOP and PI at different times. Ristic’s studies [44,45] highlighted that maximum values of PI and BOP were reached 3 months after appliance placement, followed by a decrease in these parameters 6 months after treatment began. Six studies assessed the increase of *Candida* at different times [12,13,22,23,56,57].

Twenty studies highlighted the increase of gram-positive bacteria, in particular *S. mutans* and *Lactobacillus* spp. [3,4,12,19–21,27–30,32,34,35,42,46,47,50,51,54,57]. Three studies [43,44,48] detected significant increases of gram-negative bacteria, respectively, at 3 and 6 months, followed by a decrease at 6 and 12 months. Ten studies [10,20,21,26,30,37,41,43,44,48] detected an increase in the percentage of gram-bacteria and *A. actinomycetemcomitans*. The study conducted by Alves de Souza et al. [10] revealed a significant increase in gram-species with the use of elasticomeric rings (Table 5).

**Table 5.** Conventional braces results.

Reference	PI	BOP	Microbiological analysis
Alves et al. [10]	Elastomeric rings: Value (T0) = 37.72%; value (T1) = 63.72% Steel ligatures: Value (T0) = 37.72%; value (T1) = 51.09%	Elastomeric rings: Value (T0) = 4.28%; value (T1) = 12.28% Steel ligatures: Value (T0) = 3.86%; value (T1) = 6.71%	T0 steel ligatures-elastomeric rings: *P*(Aa) = 0.3173; *P*(Tf) = 0.1797; *P*(Pg) = /; *P*(Pi) = /; *P*(Pn) = 1.000 T1 steel ligatures-elastomeric rings: *P*(Aa) = 0.3173; *P*(Tf) = 0.0039; *P*(Pg) = /; *P*(Pi) = 0.5637; *P*(Pn) = 0.0339 T0–T1 elastomeric rings: *P*(Aa) = 0.5637; *P*(Tf)<0.0001; *P*(Pg) = /; *P*(Pi) = 1,000; *P*(Pn)<0.0001 T0–T1 steel ligatures: *P*(Aa) = 0.5637; *P*(Tf) = 0.0003; *P*(Pg) = /; *P*(Pi) = /; *P*(Pn) = 0.0003
Arab et al. [12]			Salivary SM: *P*(T0) = /; *P*(T1) < 0.001; *P*(T2) < 0.001; *P*(T3) < 0.001 Lactobacillus acidophilus: *P*(T0) = /; *P*(T1) < 0.001; *P*(T2) < 0.001; *P*(T3) < 0.001 *Candida albicans*: *P*(T0) = /; *P*(T1) < 0.001; *P*(T2) < 0.001; *P*(T3) < 0.001
Arslan et al. [13]			Saliva: (T0–T1–T2–T3); *P* value <0.001 U arch: (T1–T2–T3); *P* value <0.001 L arch: (T1–T2–T3); *P* value <0.001
Forsberg et al. [19]			No. of bacteria: Elastomeric vs. steel: T2: 0.21; T3: 0.21; T4: 0.22; T5: 0.10; T6: 0.21 SM: T2: <0.01; T3: <0.01; T5: <0.001; T6: <0.01 Aerobic lactobacilli: T2: <0.05; T4: <0.001; T5: <0.05; T6: <0.01 Anaerobic lactobacili: T2: <0.01; T3: <0.01; T4: <0.01; T5: <0.001; T6: <0.001
Hägg et al. [22]	T0–T1–T2: *P* < 0.05		*Candida: P* < 0.001 after FOA with imprinted technique but not with oral rinse of pooled plaque techniques Predominant *Candida* species isolated was *C. albicans*. Number of coliform carriers after FOA –in oral rinse: *P* < 0.05, in pooled plaque: *P* < 0.05 Eight Coliform species after FOA instead of three species before FOA
Hernández-Solis et al. [23]			T0–T1: *P* < 0.001 T0: Most frequently found species *C. albicans* (8.3%); T1: *C. tropicalis* (20.0%)
Kim et al. [26]			Only significant values: *T. forsythia*: T2 vs. T3: U6: 0.013*; L6: 0.039* T2 vs. T4: U6: 0.002**; L1: 0.003**; L6: 0.012* T3 vs. T4: L1: 0.021* C. rectus: T1 vs. T2: U6: 0.007** P. nigrescens: T1 vs. T2: U6: 0.013*; L6: 0.022*
Kupietzky et al. [27]	G2 (control): mean − (SD)+ T0: 39–16; T1: 34–11 G1: mean − (SD)+ T0: 28–6; T1: 30–11 Pretest differences: *P*: 0.001		SM CFU: G2 (control): mean − (SD)+ T0: 2.5–1.2; T1: 2.8–0.9 G1: mean − (SD)+ T0: 2.9–0.9; T1: 3.3–0.8 Pretest differences: *P*: 0.09 LB CFU: G2 (control): mean − (SD)+ T0: 1.8–1.1; T1: 2.3–1.1 G1: mean − (SD)+ T0: 2.9–1.2; T1: 3.5–0.7 Pretest differences: *P*: 0.0003
Lara-Carrillo et al. [28]	O’Leary’s plaque index: *P* = 0.061		SM: T0 (M/F): *P* = 0.852; T1 (M/F): *P* = 0.575; T2 (M/F): *P* = 0.743; T3 (M/F): *P* = 0.867 LB: T0 (M/F): *P* = 0.412; T1 (M/F): *P* = 0.702; T2 (M/F): *P* = 0.428;T3 (M/F): *P* = 0.420
Lara-Carrillo et al. [29]	44.6%, M slightly greater plaque percentage (50.84%) than F (40.15%) (*P* = 0.1809)		SM: T0: 14/34 subjects had high values (>10^5^); T1: 16/34 had high values LB: T0: 7/34 subjects had high levels (>10^5^); T1: 20/34 subjects same level, although statistically significant differences were not observed in this distribution (*P* = 0.6905) Distribution of bacterial markers, plaque pH, and occult blood in saliva by gender in the study: Salivary markers: Unstimulated saliva: *P*(M/F): 0.3903/0.0026*; *P*(T0–T1): 0.4073 Stimulated saliva (ml/min): *P*(M/F): 0.0019*/0.0835; *P*(T0–T1): 0.0001* Buffer capacity: *P*(M/F): 0.0381*/0.1247; *P*(T0–T1): 0.0359* Salivary pH: *P*(M/F): 0.1672/0.7039; *P*(T0–T1): 0.0246*
Leung et al. [30]			Supragingival and subgingival plaque total DNA after appliance placement: *P* = 0.005 *Supragingival streptococci: P* = 0.0002 Buccal cells: *A. actinomycetemcomitans: P* = 0.0058
Liu et al. [32]			Total viable microflora: T0: log10 CFU ± SD: 6.94 ± 0.51; T1: log10 CFU ± SD: 7.54 ± 0.46**; T2: log10 CFU ± SD: 7.72 ± 0.36**; T3: log10 CFU ± SD: 8.07 ± 0.44** Significance: 0.0001 S: T0: log10 CFU ± SD: 5.61 ± 0.54; T1: log10 CFU ± SD: 6.34 ± 0.65**; T2: log10 CFU ± SD: 6.66 ± 0.57**; T3: log10 CFU ± SD: 6.61 ± 0.55** Significance: 0.0001 SM: T0: log10 CFU ± SD: 4.42 ± 0.62; T1: log10 CFU ± SD: 5.42 ± 0.68**; T2: log10 CFU ± SD: 5.42 ± 0.57**; T3: log10 CFU ± SD: 5.68 ± 0.65** Significance: 0.0001
Maret et al. [34]			CB was an independent risk factor for high levels of SM and LB spp. (adjusted OR: 6.65, 95% CI [1.9822.37]; 9.49, 95% CI [2.57–35.07], respectively)
Mattingly et al. [3]			T0/T1/T2 vs. T3/T4/T5: *P* < 0.01 T3 vs. T5: *P* < 0.01
Paolantonio et al. [39]	T0–T1: test: *P* < 0.001; control: *P* < 0.05 T1–T2: test: *P* < 0.05; control: *P* > 0.1 T2–T3: test: *P* > 0.1; control: *P* > 0.1 T3–T4: test: *P* < 0.001; control: *P* > 0.1 Overall T0–T4: test: *P* < 0.05; control: *P* < 0.05	T0–T1: test: *P* < 0.001; control: *P* < 0.05 T1–T2: test: *P* < 0.05; control: *P* > 0.1 T2–T3: test: *P* > 0.1; control: *P* > 0.1 T3–T4: test: *P* < 0.001; control: *P* > 0.1 Overall T0–T4: test: *P* < 0.05; control: *P* < 0.05	Mean percent of Aa+ sites: T0–T1: test: *P* < 0.001; control: – T1–T2: test: *P* > 0.1; control: *P* > 0.1 T2–T3: test: *P* > 0.1; control: – T3–T4: test: *P* < 0.001; control: – Overall T0–T4: test: *P* < 0.001; control: *P* > 0.05 Mean Aa proportion: T0–T1: test: *P* < 0.001; control: *P* > 0.05 T1–T2: test: *P* > 0.05; control: – T2–T3: test: *P* > 0.05; control: – T3–T4: test: *P* < 0.01; control: *P* > 0.1 Overall T0–T4: test: *P* < 0.001; control: *P* > 0.05
Perinetti et al. [42]	DCs: Baseline-28 days: mesial: *P* < 0.05; distal: *P* < 0.05 CCs: Baseline-28 days: mesial: *P* < 0.05; distal: *P* < 0.05 ACs: Baseline-28 days: mesial: NS; distal: NS Among groups differences: Baseline-28 days: mesial: *P* < 0.05; distal: *P* < 0.05; total: *P* < 0.01	DCs: Baseline-28 days: mesial: *P* < 0.05; distal: *P* < 0.05 CCs: Baseline-28 days: mesial: *P* < 0.05; distal: *P* < 0.05 ACs: Baseline-28 days: mesial: NS; distal: NS Among groups differences: Baseline-28 days: mesial: *P* < 0.05; distal: *P* < 0.01; total: *P* < 0.01	Aa subgingival colonization DCs: Baseline-28 days: mesial: *P* < 0.01; distal: *P* < 0.01 CCs: Baseline-28 days: mesial: *P* < 0.01; distal: *P* < 0.01 ACs: Baseline-28 days: mesial: NS; distal: NS Among groups differences: Baseline-28 days: mesial: *P* < 0.01; distal: *P* < 0.01; total: *P* < 0.01
Peros et al. [43]			SM: T0:/; T1: *P* < 0.05; T2: *P* < 0.05; T3: *P* < 0.05 LB: T0:/; T1: *P* < 0.05; T2: *P* < 0.05; T3: *P* < 0.05
Ristic et al. [44]	(mean ± SD) Incisors: T*x*: 1.281 ± 0.310; T0: 0.898 ± 0.329; T1: 1.211 ± 0.278; T2: 1.250 ± 0.336; T3: 1.219 ± 0.275 Premolars: T*x*: 0.883 ± 0.298; T0: 0.547 ± 0.314; T1: 0.984 ± 0.126; T2: 1.055 ± 0.198; T3: 1.031 ± 0.123 Molars: T*x*: 0.930 ± 0.366; T0: 0.625 ± 0.354; T1: 1.117 ± 0.277; T2: 1.109 ± 0.219; T3: 1.070 ± 0.264	(mean ± SD) Incisors: T*x*: 0.516 ± 0.416; T0: 0.266 ± 0.269; T1: 1.320 ± 0.586; T2: 1.336 ± 0.677; T3: 1.383 ± 0.453 Premolars: T*x*: 0.320 ± 0.366; T0: 0.148 ± 0.236; T1: 0.664 ± 0.379; T2: 0.672 ± 0.394; T3: 0.594 ± 0.415 Molars: T*x*: 0.234 ± 0.304; T0: 0.227 ± 0.249; T1: 0.594 ± 0.358; T2: 0.602 ± 0.347; T3: 0.547 ± 0.367	Difference between frequency of bacteria types Number determined in different periods of control: Incisors: T0–T1: *P* > 0.05; T0–T2: *P* < 0.01; T0–T3: *P* > 0.05; T1–T2: *P* < 0.05; T1–T3: *P* > 0.05; T2–T3: *P* > 0.05 Premolars: T0–T1: *P* > 0.05; T0–T2: *P* < 0.01; T0–T3: *P* > 0.05; T1–T2: *P* > 0.05; T1–T3: *P* > 0.05; T2–T3: *P* < 0.01 Molars: T0–T1: *P* < 0.05; T0–T2: *P* < 0.01; T0–T3: *P* > 0.05; T1–T2: *P* < 0.05; T1–T3: *P* > 0.05; T2–T3: *P* > 0.05 Difference between frequency findings of *P. intermedia* isolated in different periods of control: Incisors: T0–T1: *P* > 0.05; T0–T2: *P* < 0.01; T0–T3: *P* > 0.05; T1–T2: *P* < 0.05; T1–T3: *P* > 0.05; T2–T3: *P* < 0.05 Premolars: T0–T1: *P* > 0.05; T0–T2: *P* > 0.05; T0–T3: *P* > 0.05; T1–T2: *P* > 0.05; T1–T3: *P* > 0.05; T2–T3: *P* > 0.05 Molars: T0–T1: *P* > 0.05; T0–T2: *P* > 0.05; T0–T3: *P* > 0.05; T1–T2: *P* > 0.05; T1–T3: *P* > 0.05; T2–T3: *P* < 0.05
Ristic et al. [45]			Total bacterial count compared between different recording periods on incisors, premolars, and molars: Incisors: T0–T1: *P* < 0.01; T0–T2: *P* < 0.01; T1–T2: *P* < 0.01; T2–T3: *P* < 0.05 Premolars: T0–T1: *P* < 0.01; T0–T2: *P* < 0.01; T1–T2: *P* < 0.05; T2–T3: *P* > 0.05 Molars: T0–T1: *P* < 0.01; T0–T2: *P* < 0.01; T1–T2: *P* < 0.05; T2–T3: *P* > 0.05 The signiﬁcance of difference between positive ﬁndings of *Prevotella intermedia*: Incisors: *P* = 0.003; Premolars: *P* = 0.037; Molars: *P* = 0.022
Shukla et al. [47]			*P* = 0.000 (<0.05)
Shukla et al. [58]			*Candida: P* = 0 SM: *P* = 0
Sinclair et al. [4]	Plaque index: NS	Gingival index: U1: T2–T1: <0.05; L1: T2–T1: <0.05; U6: T2–T1: NS; L6: T2–T1: NS Mean: T2–T1: <0.05	S: mean: *P* < 0.01 Aa: mean: *P* < 0.05 Fusobacteria: NS Bacteroides: NS Spirochetes: NS
Sudarević et al. [48]			SM: T1–T2: NS *S. sobrinus*: T2: 2 pt
Thornberg et al. [49]			Comparison of high pathogen counts at T0 to T1, T2, T3, and T4 (significant values) *Prevotella intermedia*: T0 vs. T1: 0.0001* Tannerella forsythia: T0 vs. T1: 0.0258* Eikenella corrodens: T0 vs. T1: 0.0001*; T0 vs. T3: 0.0051*; T0 vs. T4: 0.0349* Fusobacterium nucleatum: T0 vs. T1: 0.0004*; T0 vs. T3: 0.0206*; T0 vs. T4: 0.0335* Treponema denticola: T0 vs. T1: 0.0002*; T0 vs. T3: 0.0441* Campylobacter rectus: T0 vs. T1: 0.0225*; T0 vs. T4: 0.0349*
Topaloglu et al. [50]			Means and standard deviations of SM Expressed as log10 CFU a,c*P* < 0.05, b,d*P* > 0.05 G1: SM (T0): 4.4 ± 1.1a,b; SM (T1): 4.0 ± 1.4°; SM (T2): 4.4 ± 1.1°; SM (T3): 5.2 ± 0.6b G2: SM (T0): 4.1 ± 1.0c,d; SM (T1): 4.2 ± 1.3c; SM (T2): 4.4 ± 1.0c; SM (T3): 5.5 ± 1.0d Means and standard deviations of LB spp. expressed as log10 CFU a,c*P* < 0.05, b,d*P* > 0.05: G1: LB (T0): 5.6 ± 1.2a,b; LB(T1): 5.4 ± 1.4°; LB (T2): 5.8 ± 1.3°; LB(T3): 6.6 ± 0.7b G2: LB (T0): 5.7 ± 1.0c,d; LB (T1): 5.9 ± 1.4c; LB (T2): 6.0 ± 1.1c; LB (T3): 6.3 ± 0.6d
Torlakovic et al. [51]	Plaque levels increase: NS	Prevalence of gingivitis at U1 increased from T0: 25% to T3: 74%	NS
Turkkahraman et al. [52]	Bonded bracket plaque index: G1–G2: NS T0–T1: *P* < 0.001; T0–T2: *P* < 0.001	T0 and T1: G1 ≈ G2 T2: Signiﬁcantly more bleeding in G2	Statistical comparison of bacterial counts of the groups: Total bacteria: NS Anaerobe lactobacilli: NS Longitudinal changes in bacterial counts of bonded: Total bacteria: T0–T1: *P* < 0.001; T0–T2: *P* < 0.001; T1–T2: *P* < 0.001 Anaerobe lactobacilli: T0–T1: *P* < 0.001; T0–T2: *P* < 0.001; T1–T2: *P* < 0.001 Aerobe lactobacilli: T0–T1: *P* < 0.001; T0–T2: *P* < 0.001; T1–T2: *P* < 0.001 SM: T0–T1: *P* < 0.001; T0–T2: *P* < 0.001; T1–T2: *P* < 0.001
Van Gastel et al. [20]		Banded: T1/T0 = 6.29*; bonded: T1/T0 = 3.95*	CFU ratio aerobe: anaerobe supragingival Banded: T1/T0 = 0.49*; bonded: T1/T0 = 0.51* CFU ratio aerobe: anaerobe subgingival Banded: T1/T0 = 0.25*; bonded: T1/T0 = 0.27*
Wichelhaus et al. [55]	API: Intensity 0–25: T0 (*n* = 11): number: 3 HP+: 2; T1 (*n* = 9): number: 0 HP+: 0; T2 (*n* = 11): number: 0 HP+: 0 26–35: T0 (*n* = 11): number: 0 HP+: 0; T1 (*n* = 9): number: 2 HP+: 2;T2 (*n* = 11): number: 0 HP+: 0 36–70: T0 (*n* = 11): number: 6 HP+: 6; T1 (*n* = 9): number: 5 HP+: 3; T2 (*n* = 11): number: 9 HP+: 6 71–100: T0 (*n* = 11): number: 2 HP+: 1; T1 (*n* = 9): number: 2 HP+: 0; T2 (*n* = 11): number: 2 HP+: 0	SBI: API: Intensity 0–10: T0 (*n* = 11): number: 4 HP+: 3; T1 (*n* = 9): number: 2 HP+: 2; T2 (*n* = 11): number: 2 HP+: 1 11–20: T0 (*n* = 11): number: 1 HP+: 1; T1 (*n* = 9): number: 0 HP+: 0; T2 (*n* = 11): number: 1 HP+: 1 21–50: T0 (*n* = 11): number: 5 HP+: 4; T1 (*n* = 9): number: 6 HP+: 3; T2 (*n* = 11): number: 6 HP+: 3 51–100: T0 (*n* = 11): number: 1 HP+: 1; T1 (*n* = 9): number: 1 HP+: 0; T2 (*n* = 11): number: 2 HP+: 1	LB spp.: Prevalence <10^3^: TO (*n* = 10): number: 7 HP+: 7; T1 (*n* = 9): number: 3 HP+: 2; T2 (*n* = 11): number: 2 HP+: 2 10^3^–10^4^: T0 (*n* = 10): number: 3 HP+: 2; T1 (*n* = 9): number: 5 HP+: 2; T2 (*n* = 11): number: 4 HP+: 1 >10^4^: T0 (*n* = 10): number: 0 HP+: 0; T1 (*n* = 9): number: 1 HP+: 1;T2 (*n* = 11): number: 5 HP+: 3 Streptococci: <10^5^ T0 (*n* = 10): number: 5 HP+: 4; T1 (*n* = 9): number: 6 HP+: 2; T2 (*n* = 11): number: 9 HP+: 5 10^5^–10^6^: T0 (*n* = 10): number: 3 HP+: 3; T1 (*n* = 9): number: 3 HP+: 3; T2 (*n* = 11): number: 2 HP+: 1 >10^6^: T0 (*n* = 10): number: 2 HP+: 2; T1 (*n* = 9): number: 0 HP+: 0; T2 (*n* = 11): number: 0 HP+: 0
Zheng et al. [56]			Prior to treatment: (1) *P* = 0.58143; (2) *P* = 0.000785*; (3) *P* = 0.046811*; (6) *P* = 0.318954 After 1 months: After 2 months *P* = 0.002619*; after 3 months *P* = 0.129414; after 6 months *P* = 0.64157 After 2 months: After 3 months *P* = 0.099146; after 6 months *P* = 0.009289* After 3 months: After 6 months *P* = 0.289807

/ : dental site negative; *P<0.05.

### Self-ligating braces

Eigh studies analyzed self-ligating braces (SLB) [9,14,24,37–40,54]. Two studies [14,40] revealed no differences for BOP and PI between SLB and CB, while Nalçac et al. and Uzuner et al. [54] demonstrated a worsening in SLB. Two studies considered the use of SLB with or without elastomeric rings, observing an increase in gram-concentration [24,38]. One other study [14] showed an increase of *S. mutans* and *Lactobacillus* spp. at 3 months with the use of SLB compared to controls. One study [41] showed less *S. mutans* with SLB compared to CB (Table 6).

**Table 6.** Self-ligating braces results.

Reference	PI	BOP	Microbiological analysis
Al-Anezi [9]	*P*(T0) = 0.001–*P*(T1) = 0.002	*P*(T0) = 0.125–*P*(T1) = 0.508	NS
Baka et al. [14]	Signiﬁcance between T0–T1, T1–T2, T0–T1: SL: *P*(T0–T2) = 0.000; *P*(T1–T2) = 0.000; *P*(T0–T2) = 0.000 Steel ligature: *P*(T0–T2) = 0.000; *P*(T1–T2) = 0.000; *P*(T0–T2) = 0.000 Statistical comparison of the difference in the clinical periodontal measurements between groups: Intergroup comparison: *P* value (T0–T2) = 0.091	Signiﬁcance between T0–T1, T1–T2, T0–T1: SL: *P*(T0–T2) = 0.000; *P*(T1–T2) = 0.000; *P*(T0–T2) = 0.000 Steel ligature: *P*(T0–T2) = 0.000; *P*(T1–T2) = 0.000; *P*(T0–T2) = 0.000 Statistical comparison of the difference in the clinical periodontal measurements between groups: Intergroup comparison: *P* value (T0–T2) = 0.871	Significance between T0–T2 of the bacterial counts: SM: SL: *P*(T0–T2) = 0.000; steel ligature: *P*(T0–T2) = 0.000 *S. sobrinus*: SL: *P*(T0–T2) = 0.000; steel ligature: *P*(T0–T2) = 0.000 *L. casei*: SL: *P*(T0–T2) = 0.000; steel ligature: *P*(T0–T2) = 0.000 *L. acidophilus*: SL: *P*(T0–T2) = 0.000; steel ligature: *P*(T0–T2) = 0.000 Statistical comparisons of the differences in the bacterial counts between groups: Intergroup comparison: SM: *P* = 0.787; *S. sobrinus*: *P* = 0.104; *L. casei: P* = 0.978;l L acidophilus: *P* = 0.386
Ireland et al. [24]	T0 vs. T1; T0 vs. T2: *P* < 0.001		Treponema Denticola % over total bacteria Molar band: T0 vs. T1: *P* < 0.01; T0 vs. T2: *P* < 0.05 Molar bonded tube: T0 vs. T1: *P* < 0.05; T1 vs. T3: *P* < 0.05
Nalçacı et al. [36]	G1: T0: 0.46 ± 0.06; T1: 0.60 ± 0.07; T2: 0.66 ± 0.08 T0–T1: *; T0–T2: *; L T1–T2: * G2: T0: 0.41 ± 0.05; T1: 0.60 ± 0.06; T2: 0.94 ± 0.09 T0–T1: *; T0–T2: *; T1–T2:* P value: T0: 0.511 NS; T1: 0.967 NS; T2: 0.030*	G1: T0: 0.08 ± 0.07; T1: 0.11 ± 0.11; T2: 0.13 ± 0.02 T0–T1: *; T0–T2: *; T1–T2: NS G2: T0: 0.06 ± 0.006; T1: 0.11 ± 0.008; T2: 0.21 ± 0.04 T0–T1: *; T0–T2: *; T1–T2: * P value: T0: 0.068 NS; T1: 0.092 NS; T2: 0.039*	Total bacteria: *P* value: T0: 0.877 NS; T1: 0.983 NS; T2: 0.525 NS Anaerobe lactobacilli: *P* value: T0: 0.472 NS; T1: 0.568 NS; T2: 0.738 NS Aerobe lactobacilli: *P* value: T0: 0.471 NS; T1: 0.671 NS; T2: 0.738 NS SM: *P* value: T0: 0.115 NS; T1: 0.070 NS; T2: 0.068 NS
Pandis et al. [37]	G1–G2 (T0): *P* level: NS G1–G2 (T1): *P* level: NS		Analysis of covariance for the salivary SM counts per milliliter saliva of the subjects included in the study: Log-SM: *P* = 0.033* (only significant value)
Pejda et al. [39]	FMPS: T0–T1–T2–T3: NS; G1 vs. G2: NS	FMBS during time: (*P* < 0.031) with 7.9% variability Statistically significant difference between T0 and T3 (*P* = 0.05) not influenced by type of brackets	Prevalence of AA in G2 vs. G1: *P* < 0.001. Average number of detected units of AA: G2: 10^4^–10^5^ G1 < 10^3^ Red-complex bacteria (PG, PI, TF, and TD) prevalence: NS in G1 and G2 Total count of tested species: lower in G1 (2.1 ± 1.2) than G2
Pellegrini et al. [40]			SL braces – elastomeric: Total bacteria: *P* = 0.032^*^ Oral streptococci: *P* = 0.030^*^ Number of arches exhibiting grater levels of bacteria and ATP bioluminescence in elastomeric vs. SL braces: total bacteria: T1: *P* = 0.028; T2: *P* = 0.074 Oral S: T1: *P* = 0.007; T2: *P* = 0.025 ATP bioluminescence: T1: *P* = 0.028; T2: *P* = 0.074
Uzuner et al. [53]	In SLB group PI values increased significantly (*P* < 0.05)	In SLB group GI values increased significantly (*P* < 0.05)	Comparisons of bacterial colonizations T0–T1: LB saliva: Group: 0.488; time: 0.577; group × time: 0.457 SM saliva: Group: 0.749; time: 0.341; group × time: 0.923 SM or LB colonization between the groups: NS

PI: Plaque index; BOP: bleeding on probing; SBI: sulcus bleeding index; API: interproximal plaque index. **P<0.01;  *P<0.05.

### Lingual braces

Four studies analyzed lingual braces (LB) [15,16,33,45] and three of these highlighted a worsening of PI and BOP [15,16,33]. Two studies [16,33] revealed an increase of *S. mutans* and *A. actinomycetemcomitans* after 4 weeks (Table 7).

**Table 7.** Lingual braces results.

Reference	PI	BOP	Microbiological analysis
Demling et al. [15]	Buccal sites: T0: 0.1 ± 0.2; T1: 0.1 ± 0.2 Lingual sites: T0: 0.1 ± 0.2; T1: 1.2 ± 1.1	Buccal sites: T0: 12.4 ± 8.2; T1: 14.3 ± 8.1 Lingual sites: T0: 22.2 ± 19.0; T1: 56.2 ± 31.6	AA: T0: 5 pt; T1: 4 pt PG: T0: 1 pt; T1: 2 pt
Demling et al. [16]	Maxilla: Labial: T0: 0.2 ± 0.5; T1: 0.0 ± 0.1; *P*: 0.223 Palatal: T0: 0.1 ± 0.1; T1: 0.1 ± 0.2; *P*: 0.587 Mandible: Labial: T0: 0.2 ± 0.3; T1: 0.1 ± 0.2; *P*: 0.329 Lingual: T0: 0.3 ± 0.3; T1: 1.0 ± 0.7; *P*: 0.001	Maxilla: Labial: T0: 19.9 ± 20.1; T1: 13.5 ± 13.6; *P*: 0.184 Palatal: T0: 25.2 ± 19.2; T1: 22.2 ± 18.9; *P*: 0.608 Mandible: Labial: T0: 18.1 ± 17.5; T1: 12.9 ± 16.7; *P*: 0.101 Lingual: T0: 23.4 ± 22.5; T1: 46.2 ± 23.5; *P*: 0.001	AA T0: 25% pt; T1: 35% pt PG T0, T1: 5% pt
Lombardo et al. [33]	G2: T0: 0.47 ± 0.18; T1: 0.56 ± 0.15; T2: 0.59 ± 0.16 T0–T1: *P* < 0.05; T1–T2: NS; T0–T2: *P* < 0.5 G1: T0: 0.42 ± 0.17; T1: 0.52 ± 0.25; T2: 0.43 ± 0.20 T0–T1: NS; T1–T2: NS; T0–T2: NS	G2: T0: 0.18 ± 0.13; T1: 0.22 ± 0.07; T2: 0.29 ± 0.19 T0–T1: NS; T1–T2: NS; T0: T2: *P* < 0.05 G1: T0: 0.31 ± 0.21; T1: 0.45 ± 0.17; T2: 0.33 ± 0.13 T0–T1: *P* < 0.05; T1–T2: *P* < 0.01; T0–T2: NS	SM G2: T0–T1: NS; T1–T2: NS; T0–T2: *P* < 0.05 G1: T0–T1: NS; T1–T2: NS; T0–T2: NS LB spp. : G2: T0–T1: NS; T1–T2: NS; T0–T2: NS G1: T0–T1: NS; T1–T2: NS; T0–T2: NS
Sfondrini et al. [46]		NS differences (*P* > 0.05) in the different groups at different times	Total CFU/*P* value: V–L: 4.65E + 6/0.68; V-control: 5.11E + 7/0.2; L-control: 4.64E + 7/0.41 S CFU/*P* value: V–L: 1.69E + 5/0.43; V-control: 1.11E + 6/0.96; L-control: 9.45E + 5/0.38 Anaerobe CFU/*P* value: V–L: −1.49E + 6/0.3; V-control: 3.00E + 5/0.07; L-control: 1.79E + 6/0.51

PI: Plaque index; BOP: bleeding on probing.

### Removable appliances

Six studies analyzed removable devices [11,17,18,31,49,52]. One study analyzed different interceptive removable appliances [49], demonstrating an increase in both *S. mutans* and *Lactobacillus* spp.

The invisalign study, conducted by Levrini et al. [31], revealed lower values of PI, BOP, and bacterial component at 3 months for the invisalign group.

In the two studies with thermoplastic retainers, Türköz et al. [52] showed an increase of *S. mutans* and *Lactobacillus* spp. while Farhadian et al. [18] observed that the addition of silver nanoparticles reduced the levels of *S. mutans* after 7 weeks.

In one study [11], the use of space maintainers defined an increase in BOP in the number of bacteria and in *Candida*. Furthermore, D’Ercole et al. [17] pointed out that the use of sports mouthguards produced an increase in BOP and PI (Table 8).

**Table 8.** Removable appliances results.

Reference	PI	BOP	Microbiological analysis
Arikan et al. [11]	G1: T0–T1: 0.04; T0–T2: 0.01; T0–T3: 0.34 G2: T0–T1: 0.56; T0–T2: 0.61; T0–T3: 0.27	G1: T0–T1: 0.09; T0–T2: 0.03; T0–T3: 0.001 G2: T0–T1: 0.98; T0–T2: 0.07; T0–T3: 0.05	T0: T1: Mean *Candida*: *P*(G1) = 0.68; *P*(G2) = 0.16 Total *Candida*: *P*(G1) = 0.47; *P*(G2) = 0.19 T2: Mean *Candida*: *P*(G1) = 0.003; *P*(G2) = 0.12 Total *Candida*: *P*(G1) = 0.01; *P*(G2) = 0.11 T3: Mean *Candida*: *P*(G1) = 0.00; *P*(G2) = 0.04 Total *Candida*: *P*(G1) = 0.00; *P*(G2) = 0.07
D’Ercole et al. [17]	FMPS: T0 vs. T2: *P* < 0.05*	FMBS: T0 vs. T2: *P* < 0.05*	SM: T0 vs. T1: NS; T0 vs. T2: NS; T1 vs. T2: NS
Farhadian et al. [18]			SM count: T1: *P* value = 0.586; T2: *P* value = 0.000
Levrini et al. [31]	G1 vs. G2: *P* < 0.05 G1(T0) vs. G1(T2): *P* < 0.05	G1 vs. G2: *P* < 0.05	G1 vs. G2: *P* < 0.05 G1(T0) vs. G1(T2): *P* < 0.05
Topaloglu et al. [50]			Means and standard deviations of SM expressed as log10 CFU a,c*P* < 0.05, b,d*P* > 0.05 G1: SM (T0): 4.4 ± 1.1a,b; SM (T1): 4.0 ± 1.4; SM (T2): 4.4 ± 1.1a; SM (T3): 5.2 ± 0.6b G2: SM (T0): 4.1 ± 1.0c,d; SM (T1): 4.2 ± 1.3c; SM (T2): 4.4 ± 1.0c; SM (T3): 5.5 ± 1.0d Means and standard deviations of LB expressed as log10 CFU a,c*P* < 0.05, b,d*P* > 0.05: G1: LB (T0): 5.6 ± 1.2a,b; LB (T1): 5.4 ± 1.4a; LB (T2): 5.8 ± 1.3a; LB (T3): 6.6 ± 0.7b G2: LB (T0): 5.7 ± 1.0c,d; LB (T1): 5.9 ± 1.4c; LB (T2): 6.0 ± 1.1c; LB (T3): 6.3 ± 0.6d
Türköz et al. [53]			Mean of LB at T3 (14.49 CFU/ml) higher than at T0, T1, and T2: T0–T3: *P* < 0.05 Mean of SM at T1 (43.72 CFU/ml) higher than at T0, T2, and T3: T0–T1: *P* < 0.05; T1–T2: *P* < 0.05 SM and LB scounts in saliva: NS among T0, T1, T2, and T3

PI: Plaque index; BOP: bleeding on probing.

### Other appliances

Two studies investigated other kinds of orthodontic appliances [25,56]: one fixed interceptive orthodontic appliance and one esthetic brace. In a study that analyzed fixed interceptive appliances, Ortu et al. [56] demonstrated an increase in *S. mutans* and *Lactobacillus* spp. (Table 9).

**Table 9.** Other appliances results.

Reference	PI	BOP	Microbiological analysis
Jurela et al. [25]			SM and *S. sobrinus*: NS Total bacteria counts: NS
Ortu et al. [57]			G3: T0–T1–T2: NS Group 1: T1–T2: NS; LB (T1–T2): NS; SM (T1–T2): NS Statistically significant: LB (T1–T0): *P* = 0.011; SM (T1–T0): *P* = 0.005; LB (T2–T0): *P* = 0.007; SM (T2–T0): *P* = 0.006. G2: LB (T1–T2): NS Statistically significant: LB (T2–T0): *P* = 0.006; SM (T2–T0): *P* = 0.004; LB (T1–T0): *P* = 0.01; SM (T1–T0): *P* = 0.006; SM (T1–T2): *P* = 0.03

PI: Plaque index; BOP: bleeding on probing.

## Discussion

The present systematic review agreed with the conclusions arrived at by Freitas et al. [5], which could be extended to any type of orthodontic appliance. The evidence of the selected sample was of medium-high level due to the lack of error of measurements analysis for the collection of material from oral sites. Though this lack of standardization may influence the outcomes, due to the difficulty in obtaining a high repeatability in this procedure, it would not represent a major concern for the studies’ quality. In our sample, the use of orthodontic devices resulted in an increase in oral bacterial counts in patients, with significant differences between appliance type, depending on whether they were removable or not.

Previous studies have assessed the role of biomaterials in the regulation of the oral microbiota [58]. As stated by Antonelli et al. [59], the simplest surfaces for bacteria to colonize are hard ones as mucous membranes tend to scale off and, therefore, do not guarantee a stable adhesion. The only exception to this is the tongue, which is highly colonized even if it is a mucosal surface because of the irregular surfaces of papillae [60]. Consequently, the introduction of a biomaterial into this open system creates a further retentive surface on which bacterial species are able to reproduce and where there is an increased difficulty in maintaining oral hygiene [58]. As revealed by the Øilo and Bakken [58] literature review, the presence of biomaterials results in an increase in plaque and alterations in the oral microbiota.

Thus, on the basis of these assessments, it seems reasonable to state that the grade of bacterial colonization related to orthodontic appliances is affected by the energy and roughness of the appliance surfaces, as well as their design and dimensions. This may be a key factor in efficiently performing hygiene procedures [58].

Another significant variable for microbiota alterations is the amount of time the appliance is worn in the oral cavity, with removable appliances having significantly less impact on oral bacteria than fixed appliances [61].

The quantitative alteration of the oral microbiota is related to an increase in clinical parameters, PI and BOP, which are risk indicators for oral pathologies [62].

Together with the quantitative change, there is also a qualitative variation; indeed, there is an increase in gram-positive and gram-negative more aggressive bacteria, such as: *S. mutans* and *Lactobacillus* spp. (gram-positive) and *P*. *gingivalis, Tforsythia*, and *T. denticola* (gram-negative); and these bacteria are closely associated with, respectively, enamel and dentin pathologies (e.g. demineralizations or caries) and with periodontal disease [63]. Recent papers have highlighted the complexity of periodontal disease etiology, with a special focus on the identity of bacteria which are responsible for this pathology [64–66]. Thus, authors have stated that the presence alone of specific micriobial species seems insufficient in causing gingivitis and periodontal disease, and that the change in biofilm equilibrium is another key factor in the development of these diseases [64–66]. Oral microbiota alterations registered in orthodontic patients appear to be consistent with the modifications occurring in patients with poor oral hygiene presenting gingivitis and/or periodontal diseases. In addition, orthodontic devices could represent a direct risk factor for periodontal diseases as they are often related to an increase in periodontopathogenic species [24,34,43,44,48]. However, it seems reasonable to state that the susceptibility of each subject, as well as other factors that may alter the biofilm balance, may play a key role in determining the entity of periodontal sequelae.

Even though changes in the microbial system involve all types of orthodontic appliance, more rapid modifications occur during fixed orthodontic treatment. These alterations may be recorded even 1 month after the beginning of treatment and may lead to a decrease in patients’ periodontal health perception [41]. Even so, as stated by Perinetti et al. [41], the role of subgingival bacteria in periodontal modifications needs to be evaluated together with the action of enzymes activated in response to the stimuli of orthodontic forces.

If it is true that all appliances increase the bacterial component, it is also the case that mobile devices make minor changes as they are removable and can be completely cleaned, resulting in better oral hygiene minimizing retentive artifacts. It should also be emphasized that, of these appliances, the use of mouthguards is limited to a small population and they are carried only for limited periods of time, involving a less pathogenic effect.

Less devastating results from changes in the oral microbiota emerged from studies on functional appliances and on aligners, which are used up to 22 h a day [61]. So, it seems more important to be able to remove the appliance and wash both it and the teeth rather than the length of time the device is worn.

In view of the changes in microbiota that occurred with the introduction of biomaterials into the oral cavity, and more specifically of the orthodontic devices, it would be appropriate for patients undergoing dedicated hygiene protocols to keep the oral bacterial charge under control and then to reduce the risk of the carious process and periodontal disease, as evidenced by various authors [2,67,68].

## Conclusions

- The overall evidence quality level was moderate-to-high, thus significant conclusions could be drawn.
- Orthodontic appliances significantly influence the oral microbiota, independent of appliance type.
- Significant alterations of the microbiota were registered 1 month after the start of treatment.
- Removable appliances had less impact on oral bacteria than fixed ones.
- Personalized professional and daily hygiene protocols are recommended for orthodontic patients from the beginning of treatment.

## References

[1] Alfuriji S, Alhazmi N, Alhamlan N, et al The effect of orthodontic therapy on periodontal health: a review of the literature. IntJDent. 2014;2014:585048. 10.1155/2014/585048 PMC4060421 24991214

[2] Migliorati M, Isaia L, Cassaro A, et al Efficacy of professional hygiene and prophylaxis on preventing plaque increase in orthodontic patients with multibracket appliances: a systematic review. Eur JOrthod. 2015;37(3):297–22. 25246605 10.1093/ejo/cju044

[3] Mattingly JA, Sauer GJ, Yancey JM, et al Enhancement of *Streptococcus mutans* colonization by direct bonded orthodontic appliances. JDent Res. 1983;62(12):1209–1211. 6361082 10.1177/00220345830620120601

[4] Sinclair PM, Berry CW, Bennett CL, et al Changes in gingiva and gingival flora with bonding and banding. Angle Orthod. 1987;57(4):271–278. 3479031 10.1043/0003-3219(1987)057<0271:CIGAGF>2.0.CO;2

[5] Freitas AO, Marquezan M, Nojima Mda C, et al The influence of orthodontic fixed appliances on the oral microbiota: a systematic review. Dental Press J Orthod. 2014;19(2):46–55. 10.1590/2176-9451.19.2.046-055.oar PMC4296609 24945514

[6] Moher D, Liberati A, Tetzlaff J, et al The PRISMA group. Preferred reporting items for systematic reviews and meta-analyses: the PRISMA statement. PLoS Med. 2009;6(7):e1000097. 19621072 10.1371/journal.pmed.1000097 PMC2707599

[7] Facey K, Topfer LA, Chan L. Health technology assessment (HTA) glossary. Sweden: INAHTA Secretariat, c/o SBU Stockholm; 2006.

[8] Bondemark L, Holm AK, Hansen K, et al Long-term stability of orthodontic treatment and patient satisfaction. Angle Orthod. 2007;77(1):181–191. 17029533 10.2319/011006-16R.1

[9] Al-Anezi SA Dental plaque associated with self-ligating brackets during the initial phase of orthodontic treatment: a 3-month preliminary study. J Orthod Sci. 2014;3:7–11. 24987657 10.4103/2278-0203.127550 PMC4072391

[10] Alves de Souza R, Borges de Araújo Magnani MB, Nouer DF, et al Periodontal and microbiologic evaluation of 2 methods of archwire ligation: ligature wires and elastomeric rings. Am J Orthod Dentofacial Orthop. 2008;134(4):506–512. 18929268 10.1016/j.ajodo.2006.09.067

[11] Arikan V, Kizilci E, Ozalp N, et al Effects of fixed and removable space maintainers on plaque accumulation, periodontal health, Candidal and *Enterococcus faecalis* carriage. Med Princ Pract. 2015;24(4):311–317. 26044443 10.1159/000430787 PMC5588238

[12] Arab S, Nouhzadeh Malekshah S, Abouei Mehrizi E, et al Effect of fixed orthodontic treatment on salivary flow, pH and microbial count. JDent (Tehran). 2016;13(1):18–22. 27536324 PMC4983561

[13] Arslan SG, Akpolat N, Kama JD, et al One-year follow-up of the effect of fixed orthodontic treatment on colonization by oral Candida. JOral PatholMed. 2008;37(1):26–29. 10.1111/j.1600-0714.2007.00574.x 18154574

[14] Baka ZM, Basciftci FA, Arslan U Effects of 2 bracket and ligation types on plaque retention: A quantitative microbiologic analysis with real-time polymerase chain reaction. Am J Orthod Dentofacial Orthop. 2013;144:260–267. 23910207 10.1016/j.ajodo.2013.03.022

[15] Demling A, Demling C, Schwestka-Polly R, et al Influence of lingual orthodontic therapy on microbial parameters and periodontal status in adults. Eur J Orthod. 2009;31(6):638–642. 19687149 10.1093/ejo/cjp064

[16] Demling A, Demling C, Schwestka-Polly R, et al Short-term influence of lingual orthodontic therapy on microbial parameters and periodontal status. A Preliminary Study. Angle Orthod. 2010;80:480–484. 20050740 10.2319/061109-330.1 PMC8985718

[17] D’Ercole S, Martinelli D, Tripodi D Influence of sport mouthguards on the ecological factors of the children oral cavity. BMC Oral Health. 2014;14:97. 25091394 10.1186/1472-6831-14-97 PMC4146445

[18] Farhadian N, Usefi Mashoof R, Khanizadeh S, et al *Streptococcus mutans* counts in patients wearing removable retainers with silver nanoparticles vs. those wearing conventional retainers: a randomized clinical trial. Am J Orthod Dentofacial Orthop. 2016;149(2):155–160. 26827971 10.1016/j.ajodo.2015.07.031

[19] Forsberg CM, Brattström V, Malmberg E, et al Ligature wires and elastomeric rings: two methods of ligation, and their association with microbial colonization of *Streptococcus mutans* and *Lactobacilli*. Eur J Orthod. 1991;13(5):416–420. 1748191 10.1093/ejo/13.5.416

[20] Van Gastel J, Teughels W, Quirynen M, et al Longitudinal changes in gingival crevicular fluid after placement of fixed orthodontic appliances. Am J Orthod Dentofacial Orthop. 2011;139(6):735–744. 21640879 10.1016/j.ajodo.2009.10.043

[21] Ghijselings E, Coucke W, Verdonck A, et al Long-term changes in microbiology and clinical periodontal variables after completion of ﬁxed orthodontic appliances. Orthod Craniofac Res. 2014;17(1):49–59. 23992098 10.1111/ocr.12031

[22] Hägg U, Kaveewatcharanont P, Samaranayake YH, et al The effect of fixed orthodontic appliances on the oral carriage of Candida species and Enterobacteriaceae. Eur J Orthod. 2004;26(6):623–629. 15650072 10.1093/ejo/26.6.623

[23] Hernández-Solís SE, Rueda-Gordillo F, Flota-Alcocer AD, et al Influence of orthodontic appliances on the occurrence of Candida spp. in the oral cavity. Rev Chilena Infectol. 2016;33(3):293–297. (Article in Spanish). 27598278 10.4067/S0716-10182016000300007

[24] Ireland AJ, Soro V, Sprague SV, et al The effects of different orthodontic appliances upon microbial communities. Orthod Craniofac Res. 2014;17:115–123. 24345204 10.1111/ocr.12037

[25] Jurela A, Repic D, Pejda S, et al The effect of two different bracket types on the salivary levels of *S mutans* and *S sobrinus* in the early phase of orthodontic treatment. Angle Orthod. 2013;83(1):140–145. 22765642 10.2319/030612-187.1 PMC8805532

[26] Kim SH, Choi DS, Jang I, et al Microbiologic changes in subgingival plaque before and during the early period of orthodontic treatment. Angle Orthod. 2012;82(2):254–260. 21827233 10.2319/030311-156.1 PMC8867936

[27] Kupietzky A, Majumdar AK, Shey Z, et al Colony forming unit levels of salivary *Lactobacilli* and *Streptococcus mutans* in orthodontic patients. J Clin Pediatr Dent. 2005;30(1):51–53. 16302600

[28] Lara-Carrillo E, Montiel-Bastida NM, Sánchez-Pérez L, et al Changes in the oral environment during four stages of orthodontic treatment. Korean J Orthod. 2010;40(2):95–105.

[29] Lara-Carrillo E, Montiel-Bastida NM, Sánchez-Pérez L, et al Effect of orthodontic treatment on saliva, plaque and the levels of *Streptococcus mutans* and *Lactobacillus*. Med Oral Patol Oral Cir Bucal. 2010;15(6):e924–9. 20383105 10.4317/medoral.15.e924

[30] Leung NM, Chen R, Rudney JD Oral bacteria in plaque and invading buccal cells of young orthodontic patients. Am J Orthod Dentofacial Orthop. 2006;130(6):698.e11-8. 10.1016/j.ajodo.2006.05.028 17169727

[31] Levrini L, Mangano A, Montanari P, et al Periodontal health status in patients treated with the Invisalign® system and fixed orthodontic appliances: a 3 months clinical and microbiological evaluation. Eur J Dent. 2015;9(3):404–410. 26430371 10.4103/1305-7456.163218 PMC4569994

[32] Liu J, Bian Z, Fan MW, et al Typing of *mutans streptococci* by arbitrarily primed PCR in patients undergoing orthodontic treatment. Caries Res. 2004;38(6):523–529. 15528906 10.1159/000080581

[33] Lombardo L, Ortan YÖ, Gorgun Ö, et al Changes in the oral environment after placement of lingual and labial orthodontic appliances. Prog Orthod. 2013;14:28. 24326120 10.1186/2196-1042-14-28 PMC4384913

[34] Maret D, Marchal-Sixou C, Vergnes JN, et al Effect of fixed orthodontic appliances on salivary microbial parameters at 6 months: a controlled observational study. J Appl Oral Sci. 2014;22(1):38–43. 24626247 10.1590/1678-775720130318 PMC3908763

[35] Miura KK, Ito IY, Enoki C, et al Anticariogenic effect of fluoride-releasing elastomers in orthodontic patients. Braz Oral Res. 2007;21(3):228–233. 17710288 10.1590/s1806-83242007000300007

[36] Nalçacı R, Özat Y, Çokakoğlu S, et al Effect of bracket type on halitosis, periodontal status, and microbial colonization. Angle Orthodont. 2014;84:479–485. 24188122 10.2319/061913-461.1 PMC8667509

[37] Pandis N, Papaioannou W, Kontou E, et al Salivary *Streptococcus mutans* levels in patients with conventional and self-ligating brackets. Eur J Orthod. 2010;32(1):94–99. 19474229 10.1093/ejo/cjp033

[38] Paolantonio M, Festa F, di Placido G, et al Site-specific subgingival colonization by *Actinobacillus actinomycetemcomitans* in orthodontic patients. Am J Orthod Dentofacial Orthop. 1999;115(4):423–428. 10194288 10.1016/s0889-5406(99)70263-5

[39] Pejda S, Varga ML, Milosevic SA, et al Clinical and microbiological parameters in patients with self-ligating and conventional brackets during early phase of orthodontic treatment. Angle Orthodont. 2013;83:133–139. 22765511 10.2319/010412-8.1 PMC8805529

[40] Pellegrini P, Sauerwein R, Finlayson T, et al Plaque retention by self-ligating vs elastomeric orthodontic brackets: quantitative comparison of oral bacteria and detection with adenosine triphosphate-driven bioluminescence. Am J Orthod Dentofacial Orthop. 2009;135:426.e1-426.e9. 10.1016/j.ajodo.2008.12.002 19361723

[41] Perinetti G, Paolantonio M, Serra E, et al Longitudinal monitoring of subgingival colonization by *Actinobacillus actinomycetemcomitans*, and crevicular alkaline phosphatase and aspartate aminotransferase activities around orthodontically treated teeth. J ClinPeriodontol. 2004;31(1):60–67. 10.1111/j.0303-6979.2004.00450.x 15058376

[42] Peros K, Mestrovic S, Anic-Milosevic S, et al Salivary microbial and nonmicrobial parameters in children with fixed orthodontic appliances. Angle Orthodont. 2011;81(5):901–906. 21534725 10.2319/012111-44.1 PMC8916182

[43] Ristic M, Vlahovic Svabic M, Sasic M, et al Clinical and microbiological effects of ﬁxed orthodontic appliances on periodontal tissues in adolescents. Orthod Craniofac Res. 2007;10:187–195. 17973685 10.1111/j.1601-6343.2007.00396.x

[44] Ristic M, Vlahovic Svabic M, Sasic M, et al Effects of ﬁxed orthodontic appliances on subgingival microﬂora. Int J Dent Hyg. 2008;6:129–136. 18412726 10.1111/j.1601-5037.2008.00283.x

[45] Sfondrini MF, Debiaggi M, Zara F, et al Influence of lingual bracket position on microbial and periodontal parameters in vivo. JAppl Oral Sci. 2012;20(3):357–361. 22858704 10.1590/S1678-77572012000300011 PMC3881777

[46] Shukla C, Maurya RK, Singh V, et al Evaluation of changes in *Streptococcus mutans* colonies in microflora of the Indian population with fixed orthodontics appliances. DentResJ (Isfahan). 2016;13(4):309–314. 10.4103/1735-3327.187876 PMC4993057 27605987

[47] Sudarević K, Jurela A, Repić D, et al Oral health changes during early phase of orthodontic treatment. Acta Clin Croat. 2014;53:399–404. 25868306

[48] Thornberg MJ, Riolo CS, Bayirli B, et al Periodontal pathogen levels in adolescents before, during, and after fixed orthodontic appliance therapy. Am J Orthod Dentofacial Orthop. 2009;135(1):95–98. 19121507 10.1016/j.ajodo.2007.02.057

[49] Topaloglu-Ak A, Ertugrul F, Eden E, et al Effect of orthodontic appliances on oral microbiota—6 month follow-up. JClin Ped Dent. 2011;35(4):433–436. 10.17796/jcpd.35.4.61114412637mt661 22046705

[50] Torlakovic L, Paster BJ, Øgaard B, et al Changes in the supragingival microbiota surrounding brackets of upper central incisors during orthodontic treatment. Acta Odontol Scand. 2013;71(6):1547–1554. 24180590 10.3109/00016357.2013.776107

[51] Turkkahraman H, Sayın MO, Bozkurt FY, et al Archwire ligation techniques, microbial colonization, and periodontal status in orthodontically treated patients. Angle Orthodont. 2005;75:231–236. 15825788 10.1043/0003-3219(2005)075<0227:ALTMCA>2.0.CO;2

[52] Türköz C, Canigür Bavbek N, Kale Varlik S, et al Influence of thermoplastic retainers on *Streptococcus mutans* and *Lactobacillus* adhesion. Am J Orthod Dentofacial Orthop. 2012;141(5):598–603. 22554754 10.1016/j.ajodo.2011.11.021

[53] Uzuner FD, Kaygisiz E, Cankaya ZT Effect of the bracket types on microbial colonization and periodontal status. Angle Orthodont. 2014;84:1062–1067. 24731062 10.2319/111813-844.1 PMC8638507

[54] Wichelhaus A, Brauchli L, Song Q, et al Prevalence of Helicobacter pylori in the adolescent oral cavity: dependence on orthodontic therapy, oral flora and hygiene. J Orofac Orthop. 2011;72(3):187–195. 21744197 10.1007/s00056-011-0024-5

[55] Zheng Y, Li Z, He X Influence of fixed orthodontic appliances on the change in oral Candida strains among adolescents. JDent Sci. 2014;11(1):17–22. 10.1016/j.jds.2014.02.001 PMC6395155 30894940

[56] Ortu E, Sgolastra F, Barone A, et al Salivary *Streptococcus Mutans* and *Lactobacillus spp*. levels in patients during rapid palatal expansion. Eur J Paediatr Dent. 2014;15(3):271–274. 25306143

[57] Shukla C, Maurya R, Singh V, et al Evaluation of role of fixed orthodontics in changing oral ecological flora of opportunistic microbes in children and adolescent. J Indian Soc Pedod Prev Dent. 2017;35(1):34–40. 28139480 10.4103/0970-4388.199226

[58] Øilo M, Bakken V Biofilm and dental biomaterials. Materials. 2015;8:2887–2890.

[59] Antonelli G, Clementi M, Pozzi G, et al Principi di microbiologia medica. II ed. Milano: Casa Editrice Ambrosiana; 2012.

[60] Hall MW, Singh N, Ng KF, et al Inter-personal diversity and temporal dynamics of dental, tongue, and salivary microbiota in the healthy oral cavity. NPJ Biofilms Microbiomes. 2017 1 26;3:2. 28649403 10.1038/s41522-016-0011-0 PMC5445578

[61] Rossini G, Parrini S, Castroflorio T, et al Periodontal health during clear aligners treatment: a systematic review. Eur J Orthod. 2015;37(5):539–543. 25548145 10.1093/ejo/cju083

[62] Lindhe J, Lang NP Parodontologia clinica e implantologia orale. IV ed. Milano: Edi-Ermes; 2016.

[63] Wong BK, McGregor NR, Butt HL, et al Association of clinical parameters with periodontal bacterial haemolytic activity. JClin Periodontol. 2016;43(6):503–511. 27105613 10.1111/jcpe.12554

[64] Larsen T, Fiehn NE Dental biofilm infections – an update. APMIS. 2017;125:376–384. 28407420 10.1111/apm.12688

[65] Vieira Colombo AP, Magalhães CB, Hartenbach FA, et al Periodontal-disease-associated biofilm: a reservoir for pathogens ofmedical importance. Microb Pathog. 2016;94:27–34. 26416306 10.1016/j.micpath.2015.09.009

[66] Sanz M, Beighton D, Curtis MA, et al Role of microbial biofilms in the maintenance of oral health and in the development of dental caries and periodontal diseases. Consensus report of group 1 of the Joint EFP/ORCA workshop on the boundaries between caries and periodontal disease. J Clin Periodontol. 2017;44(Suppl 18):S5–S11. 28266109 10.1111/jcpe.12682

[67] Lucchese A, Gherlone E Prevalence of white-spot lesions before and during orthodontic treatment with fixed appliances. EurJOrthod. 2013;35:664–668. 10.1093/ejo/cjs070 23045306

[68] Hamdan AM, Maxfield BJ, Tufekci E, et al Preventing and treating white-spot lesions associated with orthodontic treatment: a survey of general dentists and orthodontists. J Am Dent Assoc. 2012;143(7):777–783. 22751981 10.14219/jada.archive.2012.0267

